# Efficacy of befotertinib in non-small cell lung cancer harboring uncommon compound EGFR mutations G719X and S768I: a case report

**DOI:** 10.3389/fonc.2024.1370666

**Published:** 2024-04-04

**Authors:** Zhedong Zhang, Yu Huang, Haihua Gu, Lufeng Zhao, Baiqin Zhao

**Affiliations:** Department of Thoracic Surgery, The Second Affiliated Hospital, School of Medicine, Zhejiang University, Hangzhou, Zhejiang, China

**Keywords:** NSCLC, EGFR tyrosine kinase inhibitors, EGFR G719X/S768I, next-generation sequencing, case report

## Abstract

The discovery of epidermal growth factor receptor (EGFR) somatic mutations and the availability of tyrosine kinase inhibitors (TKIs) as targeted therapies have transformed the treatment landscape for advanced non-small cell lung cancer (NSCLC). *p.*G719X and *p.*S768I mutations, often present in the form of complex mutations, are considered rare. This study firstly reported the treatment outcome of a locally advanced unresectable NSCLC patient with a rare complex EGFR *p.*G719X/*p.*S768I mutations who received befotertinib. After treatment, the patient achieved partial response (PR), and no severe adverse events were observed. This case report supported befotertinib as a promising treatment option for advanced NSCLC patients with the rare *p.*G719X/*p.*S768I complex mutations.

## 1 Introduction

Lung cancer stands as the foremost cause of cancer-related mortality on a global scale, with non-small cell lung cancer (NSCLC) comprising roughly 80-85% of all cases (1). At present, epidermal growth factor receptor tyrosine kinase inhibitors (EGFR-TKIs) are unanimously regarded as the preferred frontline treatment for EGFR-positive NSCLC (2). Due to the rarity of compound EGFR mutations, high mutational heterogeneity, and limited data reports, treatment strategies and clinical outcomes for patients with comorbidities remain controversial (3). Therefore, we reported a case of lung adenocarcinoma carrying rare compound EGFR mutations (EGFR exon *p.*G719X and *p.*S768I) and treated with befotertinib, a third-generation (3G) EGFR-TKI.

## 2 Case presentation

**Figure 1:**
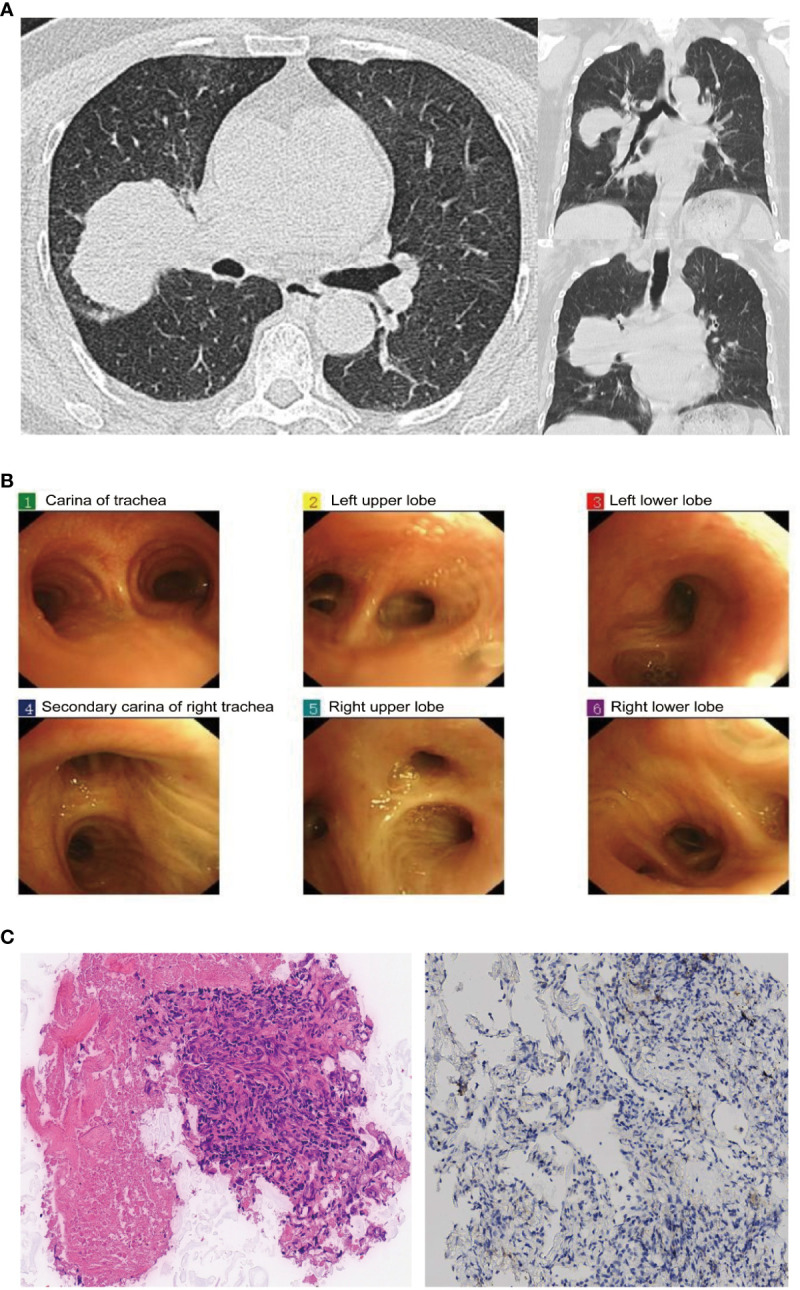
Chest computed tomography (CT), bronchoscopy, and pathological results at admission: **(A)** Chest CT scan reveals a 60×51mm mass in the right upper lung lobe. **(B)** Bronchoscopy examination does not show any clear intratracheal abnormalities. **(C)** Pathological examination of the biopsy specimen (H&E stain, original magnification, ×200 times) and PD-L1 immunohistochemical staining (×100 times).

In June 2023, a 70-year-old Chinese female complained of a pulmonary mass discovered during a routine health examination one month prior. She had never smoked and had no relevant medical, family history, or psychosocial issues. High-resolution computed tomography (HRCT) scans indicated a solid lesion spanning approximately 60×51 mm in the right upper lobe (RUL) of the lung, with shallow lobulation, bronchial obstruction, and concurrent obstructive pneumonia, including the middle lobe. Enlarged lymph nodes of the 4R group were noted within the mediastinum, with a short-axis diameter of approximately 1.6 cm (**Figure 1A**). Abdominal color ultrasound, cranial magnetic resonance imaging, and emission computed tomography did not indicate metastasis in other anatomical sites. Bronchoscopy revealed no discernible abnormalities (**Figure 1B**). Laboratory tests indicated elevated levels of carcinoembryonic antigen (CEA) at 14.5 ng/ml and cytokeratin-19 fragment (Cyfra 21-1) at 6.3 ng/ml. CT-guided lung biopsy confirmed the presence of adenocarcinoma within the RUL mass. The patient was diagnosed with stage IIIB adenocarcinoma of NSCLC (cT3N2M0) suitable for surgical resection according to the 8th edition of the American Joint Committee on Cancer (AJCC) tumor-node-metastasis (TNM) staging system.

On June 21, 2023, the patient was scheduled to undergo video-assisted thoracoscopic surgery for curative resection of the right lung cancer. Intraoperatively, exploration revealed the following findings: The tumor was located in the RUL, characterized by its hardness and indistinct boundaries. The pleural surface and the pleura of the right middle lobe (RML) showed significant retraction. The tumor invaded the RML and showed dense adhesions without any space between it and the right middle lobe pulmonary artery. Noticeably enlarged lymph nodes were observed in the 4R/10R/11R groups, densely adhering to the upper pulmonary vein, the main branch of the right lower lobe pulmonary artery, and the pre-hilar portion of the upper pulmonary vein. Intraoperatively, it was determined that the tumor was unresectable at stage IIIB. Therefore, a sampling of lymph nodes in the 4R/10R/11R groups and a biopsy of the lung tumor were performed, which indicated adenocarcinoma (**Figure 1C**), with immunohistochemistry results as follows: TTF-1 (SPT24) (-), NapsinA (+), P40 (-), P63 (-), Ki-67 (10%+), CK5/6 (-), and PD-L1 immunohistochemical testing yielded negative results. No tumor involvement was detected in the 4R/10R/11R lymph nodes.

**Figure 2:**
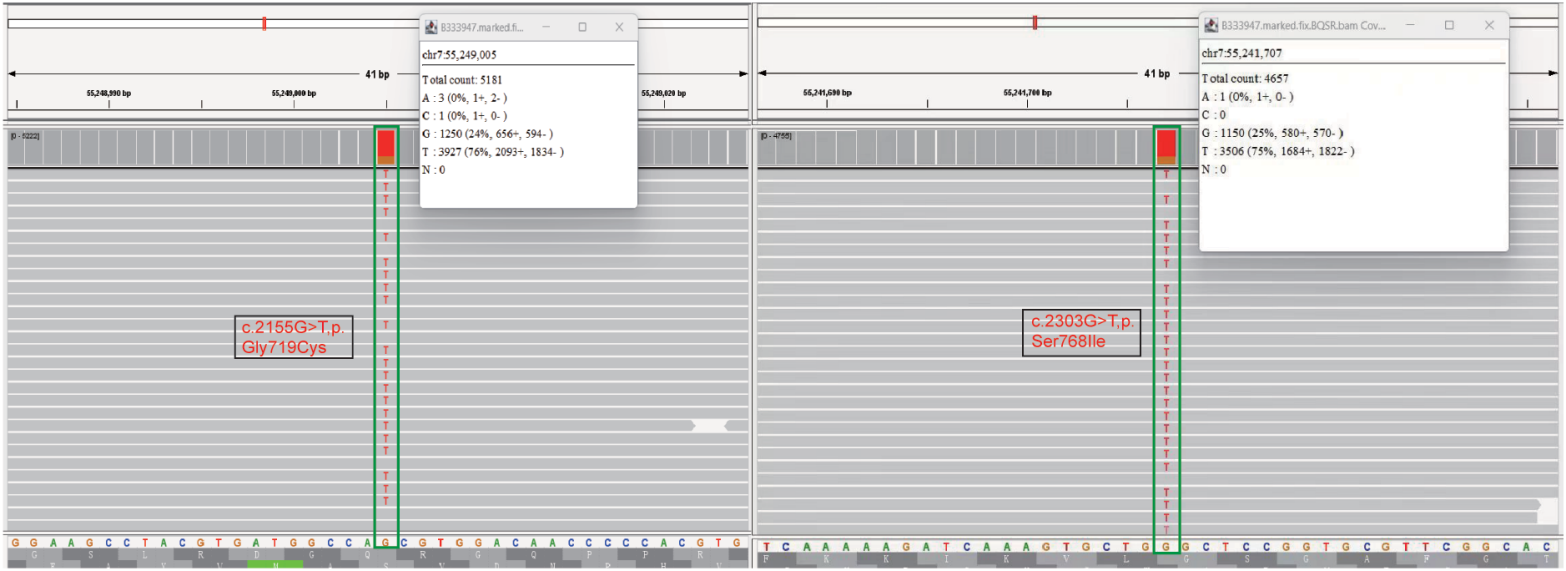
Identification of *p.*G719X (c.2155G>T) in EGFR exon 18 and *p.*S768I (c.2303G>T) in exon 20 by next-generation sequencing.

**Figure 3:**
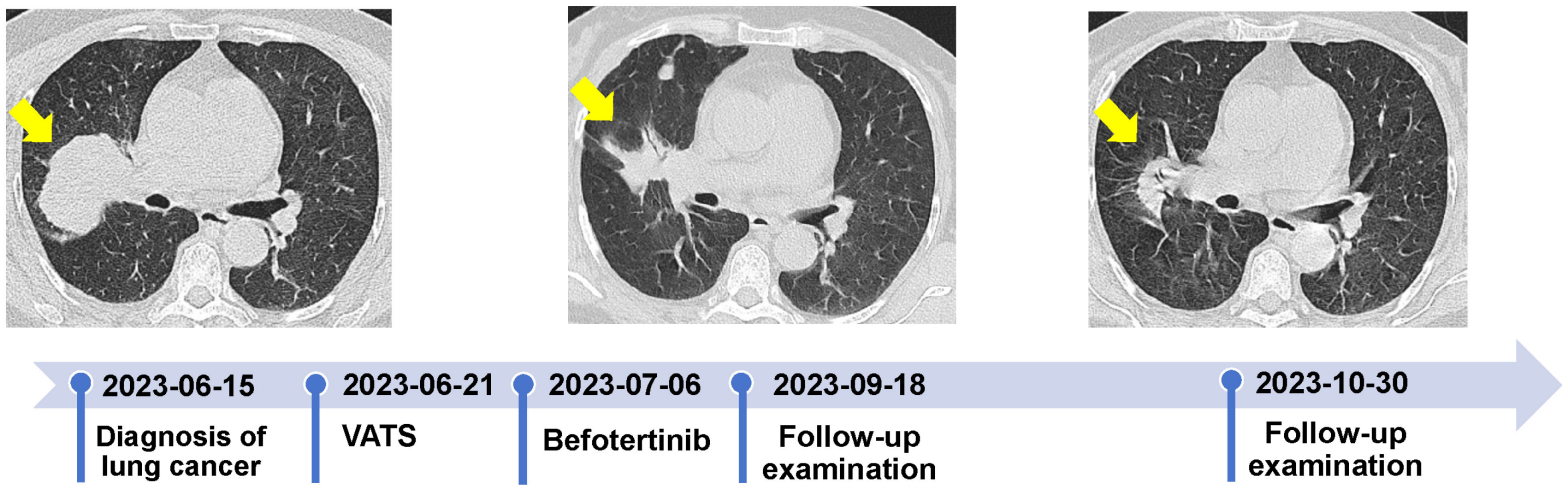
Clinical course of the patient.

Following discussions within a multidisciplinary team, the patient’s post-operative physical performance status was classified as PS2. Next-generation sequencing (NGS) analysis using a panel (Illumina nova 6000 platform) covering 520 cancer-related genes revealed missense mutations in exon 18 [EGFR *p.*G719X (c.2155G>T, *p.*Gly719Cys)] and 20 [EGFR *p.*S768I (c.2303G>T, *p.*Ser768Ile)] (**Figure 2**). No mutations were detected in ALK, ROS1, RET, KRAS, NRAS, BRAF, PIK3CA, NTRK, HER-2, and MET. On July 6, 2023, she was initiated and treated with befotertinib (75 mg/day with a gradual increase in dosing for three weeks, followed by an escalation to 100 mg/day). On September 18, 2023, an HRCT scan indicated a reduction in the size of the RUL tumor, measuring approximately 40 mm × 28 mm, the scan indicated a reduction in the size of the RUL tumor, measuring approximately 40 mm × 28 mm; the patient achieved partial response (PR). The levels of CEA and Cyfra 21-1 decreased to 6.3 ng/ml and 1.6 ng/ml, respectively. One month later, the RUL tumor was further reduced to approximately 35×25 mm (**Figure 3**). The patient tolerated oral befotertinib treatment well, had no dissatisfaction, and cooperated actively, experiencing only local rash (Grade 1 according to the National Cancer Institute Common Terminology Criteria for Adverse Events 5.0), which improved with symptomatic treatment. Currently, the patient continues befotertinib treatment. Due to personal reasons, recent scheduled chest CT scans and tumor marker assessments have been missed. Follow-up consultations are being conducted via telephone.

## 3 Discussion

In published case reports of lung adenocarcinoma with *p.*G719X and *p.*S768I mutations, different treatment approaches were employed, including oral gefitinib (first-generation, 1G), afatinib (second-generation, 2G), and osimertinib (3G) (**Supplementary Table 1**). In patients with the complex dual mutation of *p.*G719X and *p.*S768I, the objective response rate (ORR) was only 53% when treated with gefitinib (4). However, Do et al. reported a case of metastatic non-small cell lung cancer with the rare *p.*G719X and *p.*S768I compound mutation, where the patient benefitted from first-line treatment with gefitinib, with a response duration exceeding 44 months, demonstrating a durable response to the first-generation (1G) EGFR-TKI (5). For second-generation (2G) EGFR-TKI treatment, NSCLC patients with *p.*G719X, *p.*S768I, and *p.*G719X+*p.*S768I mutations have demonstrated favorable responses to afatinib, with the highest reported objective response rate (ORR) reaching as high as 77.1%-100% (6). Moreover, lung adenocarcinoma patients carrying EGFR gene mutations *p.*G719X and *p.*S768I have successfully utilized afatinib treatment, resulting in a prolonged progression-free survival (PFS) of up to 17 months (7). Although the Food and Drug Administration (FDA) has approved afatinib for the treatment of non-common EGFR mutations, including *p.*G719X, *p.*S768I, and *p.*L861Q, the efficacy of EGFR-TKI therapy in patients with rare mutations remains unclear (8). Currently, the impact of 3G EGFR-TKIs is still being explored. Since the FDA approved osimertinib as a first-line therapy for advanced NSCLC with EGFR mutations, there have been a few case reports of patients with this complex mutation type receiving osimertinib treatment. Currently, a study is underway to evaluate the 5-year efficacy and safety of osimertinib as an adjuvant therapy for EGFR rare sensitizing mutations, including *p.*G719X, *p.*L861Q, and/or *p.*S768I (TARGET, NCT05526755) (9). Befotertinib, approved by the National Medical Products Administration (NMPA) formally as a 3G EGFR-TKI for treating locally advanced or metastatic NSCLC, was derived from the molecular structure of osimertinib, with structural optimization achieved by substituting a trifluoromethyl group for a methyl group on the indole nitrogen atom. Simultaneously, befotertinib exhibits enhanced clinical efficacy over osimertinib in first-line patients with advanced-stage disease (median progression-free survival 22.1 months vs. 8.2 months) (10, 11). Given the current literature reporting the efficacy of 3G EGFR-TKI drugs for this rare mutation, alongside befotertinib’s favorable outcomes in locally advanced or metastatic NSCLC and reported adverse reactions to afatinib, befotertinib is chosen as a treatment option. Our case demonstrated that befotertinib elicited a positive response in advanced NSCLC patients with rare *p.*G719X and *p.*S768I complex mutations, presenting a new treatment option for this rare complex mutation.

Adverse events related to 1G and 2G EGFR TKIs are frequently observed and can substantially impact patients’ quality of life. Common examples of these adverse effects include skin rashes, diarrhea, mucositis, and stomatitis. Compared to the previous two generations, 3G EGFR TKIs significantly overcome these adverse events (12). For the treatment of this rare mutation with EGFR TKIs, only three case reports have described the adverse reactions. These reports include diarrhea (Grade 2), rash (Grade 2), stomatitis (Grade 2), nausea (Grade 3), diarrhea (Grade 2), peritonitis with afatinib, and no adverse reactions with osimertinib (**Supplementary Table 1**). Befotertinib, as an I-class 3G EGFR-TKI, has been optimized in its molecular structure design. N-trifluoromethyl is more stable *in vivo* than a methyl group, which can prevent the metabolism that generates AZ5104 and reduce gastrointestinal adverse reactions such as diarrhea (13). In this case, the patient developed a rash (Grade 1) only on the forearm during treatment.

The strength of this study was that we presented a rare case of lung cancer with a unique combination of EGFR *p.*G719X and *p.*S768I mutations, which showed a positive response to single-agent treatment with befotertinib. This is the first report of a favorable response to the 3G EGFR-TKI befotertinib as a monotherapy. However, there are some limitations, are such as large population and long-term follow-up studies being required to offer more persuasive evidence.

## 4 Conclusions

Befotertinib was effective and safe in the treatment of NSCLC patients with rare *p.*G719X/*p.*S768I complex mutations and might be a promising therapeutic strategy. Moreover, further clinical trials and in-depth investigation of the molecular mechanisms are necessary.

## Data availability statement

The original contributions presented in the study are included in the article/**Supplementary Material**. Further inquiries can be directed to the corresponding author.

## Ethics statement

The studies involving humans were approved by The second affiliated hospital, school of medicine, Zhejiang University committee. The studies were conducted in accordance with the local legislation and institutional requirements. The participants provided their written informed consent to participate in this study. Written informed consent was obtained from the individual(s) for the publication of any potentially identifiable images or data included in this article.

## Author contributions

ZZ: Data curation, Formal analysis, Investigation, Project administration, Software, Visualization, Writing – original draft. YH: Investigation, Project administration, Writing – review & editing. HG: Investigation, Project administration, Writing – review & editing. LZ: Investigation, Project administration, Writing – review & editing. BZ: Conceptualization, Investigation, Project administration, Resources, Supervision, Writing – review & editing.
