# Supplementary material for: Efficacy of befotertinib in non-small cell lung cancer harboring uncommon compound EGFR mutations G719X and S768I: a case report
Source: Front Oncol. 2024 Apr 4;14:1370666. doi: 10.3389/fonc.2024.1370666 (PMC11024360; doi:10.3389/fonc.2024.1370666)
Supplement: Supplementary file 1 [file Table_1.docx]

| **Table S1** Published cases of EGFR G719X/S768I complex mutations in NSCLC and their response to therapy | | | | | | | | |
| --- | --- | --- | --- | --- | --- | --- | --- | --- |
| **Authors/Report year** | **Age/Sex** | **Race** | **Smoker** | **Stage** | **Site of distant metastases** | **Treatment** | **Adverse reaction** | **Outcome** |
| Svaton et al./2015^[1](#_ENREF_1)^ | 63/F | Non-Asian | No | T1N2M1b, IVB | PUL, BON, LIV | Gefitinib | NA | PR (8 months) |
| Watanabe et al./2018[^2^](#_ENREF_2) | 72/F | Asian | NA | T4N3M0, IIIB | - | Afatinib | diarrhea (G2), rash (G2), stomatitis (G2), and nausea (G3) | PR (12 months) |
| Cai et al./2020[^3^](#_ENREF_3) | 56/F | Asian | No | T1N0M0, IA | - | Osimertinib | No | PR (31 months) |
| Kutsuzawa et al./2020[^4^](#_ENREF_4) | 67/M | Non-Asian | Yes | T1N2M0, IIIA | - | Afatinib | diarrhea (G2), peritonitis (G2) | PR (17 months) |
| Masuda et al./2020[^5^](#_ENREF_5) | 77/M | Asian | Yes | T4N3M1c, IVB | PUL, BON, PLE, ADR, BRA | Afatinib | No | PR (38 months) |
| Laguado et al./2022[^6^](#_ENREF_6) | 65/F | Non-Asian | NA | T2N3M1, IVB | BON, PUL | Osimertinib | NA | PD (8 months) |
| Do et al./2023[^7^](#_ENREF_7) | 68/M | Asian | NA | T4NxM1, IVA | PUL | Gefitinib | No | PR (44 months) |
| Morimoto et al./2023[^8^](#_ENREF_8) | 72/M | Asian | Yes | T4NxM1c, IVB | BRA | Osimertinib | No | PR (15 months) |
| Abbreviations: F, female; M, male; PUL, pulmonary; Bon, bone; LIV, liver; PLE, pleural; ADR, adrenal; BRA, brain; NA, not available; G, grade; PR, partial response; PD, progressive disease. | | | | | | | | |

**References**

1. Svaton M, Pesek M, Chudacek Z, et al. Current two EGFR mutations in lung adenocarcinoma -  case report. *Klinicka onkologie : casopis Ceske a Slovenske onkologicke spolecnosti* 2015;28:134-137.

2. Watanabe M, Oizumi S, Kiuchi S, et al. The Effectiveness of Afatinib in a Patient with Advanced Lung Adenocarcinoma Harboring Rare G719X and S768I Mutations. *Internal medicine (Tokyo, Japan)* 2018;57:993-996.

3. Cai Y, Wang Y, Sun J, et al. Successful treatment of a patient with NSCLC carrying uncommon compound EGFR G719X and S768I mutations using osimertinib: A case report. 2020;48:300060520928793.

4. Kutsuzawa N, Takahashi F, Tomomatsu K, et al. Successful Treatment of a Patient with Lung Adenocarcinoma Harboring Compound EGFR Gene Mutations, G719X and S768I, with Afatinib. *The Tokai journal of experimental and clinical medicine* 2020;45:113-116.

5. Masuda T, Sunaga N. Successful afatinib rechallenge in a patient with non-small cell lung cancer harboring EGFR G719C and S768I mutations. *Journal of international medical research* 2020;11:2351-2356.

6. Zapata Laguado M, Zuluaga A, Parra Medina R, et al. Two Unusual Mutations in the Epidermal Growth Factor Receptor Gene in a Patient With Lung Adenocarcinoma. *Cureus* 2022;14:e22372.

7. Do KH, Le DT. Prolonged response to first-generation tyrosine kinase inhibitor in a metastatic non-small cell lung cancer harbouring complex G719X and S768I mutations: A case report from Vietnam and literature review. *Respirology case reports* 2023;11:e01131.

8. Morimoto T, Yamasaki K. A rare case of double primary lung adenocarcinomas with uncommon complex EGFR G719X and S768I mutations and pleomorphic carcinoma. *Thoracic cancer*  2023;14:2981-2984.
